# On characteristics of *K*_0_ value and shear behaviour of loess using triaxial test

**DOI:** 10.1038/s41598-023-42248-1

**Published:** 2024-05-29

**Authors:** Xin Liu, Xinyu Xu, Liang Huang, Xiao Wei, Hengxing Lan

**Affiliations:** 1 School of Geological Engineering and Geomatics, Chang’an University, Xi’an 710054, China; 2 Research Center of Coastal and Urban Geotechnical Engineering, Zhejiang University, Hangzhou, 310058 China; 3 LREIS, Institute of Geographic Sciences and Natural Resources Research, Chinese Academy of Sciences, Beijing, 100101 China

**Keywords:** Natural hazards, Solid Earth sciences

## Abstract

Compared with conventional soils, such as sand and clay, little knowledge on the coefficient of lateral earth pressure at-rest (*K*_0_) has been established for loess in the current literature. This paper presents an experimental investigation on *K*_0_ of compacted loess and the associated impacts on undrained shear behaviour. By adopting a *K*_0_ consolidation module in the triaxial system, the *K*_0_ stress state for loess samples was achieved through a unique feedback control. During the *K*_0_ consolidation, the deviatoric stress (*q*) increases progressively with the premise that the volumetric strain (*ε*_v_) of the sample equals to the axial strain (*ε*_a_). The results show that the *K*_0_ value of compacted loess is in a range of 0.28 to 0.53, which is dependent on the packing density and the clay content. A distinguishable decrease of *K*_0_ was found in the course of *K*_0_ consolidation for the loosely compacted loess sample, whereas a similar trend was not observed in the dense sample. In the undrained shear stage, all loess specimens revealed contractive response in the stress path (*q*-*p*’) diagram, which can be quantified by a modified collapsibility index (*I*_c_). The index is consistently higher for the *K*_0_ consolidated loess samples than for the isotropic ones. The experimental results indicate a strong impact of the initial stress state on the shear behaviour of compacted loess.

## Introduction

**Figure 1:**
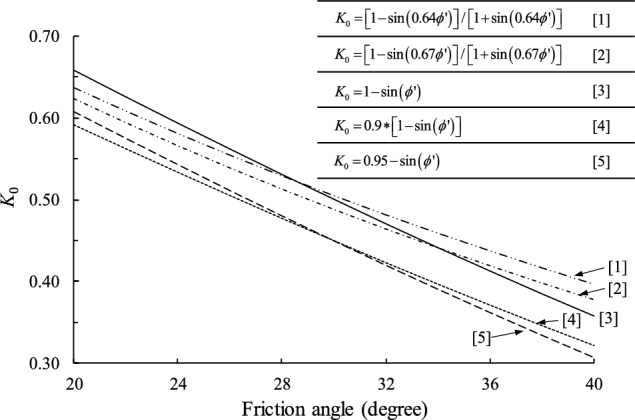
Comparison of the commonly used methods for *K*_0_ calculation.

Below a level ground, the horizontal stress (*σ’*_h_) is not the same as the vertical overburden stress (*σ’*_v_). A common approach to characterize such differences of stress is to use a coefficient of lateral earth pressure at-rest, *K*_0_ = *σ’*_h_/*σ’*_v_, which serves as a key parameter in an expand range of geotechnical designs, such as embankments, retaining structures, etc. Extensive studies have been conducted to estimate the *K*_0_ of sand and clay by using empirical or semi-empirical relationships^1^, yet among which the level of confidence in a universal application is not high. Several relationships in the literature to predict *K*_0_ by using the friction angle are compared in Fig. 1^2–6^. It is clearly seen that discrepancies exist. For a given friction angle of 36°, for instance, the* K*_0_ value is determined as 0.44 using the proposed equation by Federico et al.^2^, whereas it is 0.36 using the equation by Brooker and Ireland^6^.

As far as loess is of concern, it exhibits a wider distribution of particle size than the clean sand or clay^7^. Recent advances of engineering projects in the Chinese Loess Plateau have prompted massive earth-filled works on loess^8–12^. Yet, compared with the extensively investigated conventional soils, a lack of experimental evidence impedes understanding of *K*_0_ for loess. Current practice to estimate the *K*_0_ value of loess still mainly use the routine methods for sand from the textbook^8–12^.

Furthermore, to get a thorough understanding of the undrained response of compacted loess is crucial in the instability analysis of earth structures. Previous investigations often focus on the conventional triaxial test method^13^, where soil samples are consolidated under an isotropic stress state. To replicate the *K*_0_ stress state in a triaxial test, a soil sample needs to be consolidated anisotropically with proper control of the stress and deformation of the specimen, through which the lateral constraint of the soil can be fulfilled^14–16^. Existing research for granular soils in the literature indicate that anisotropic consolidation can significantly alter the shearing responses of soils including the shear strength and the failure modes as compared with the soils subjected to an isotropic loading condition^17–19^. By contrast, the impact of *K*_0_ consolidation on the shear response of loess has been far less extensively studied and the associated knowledge is rather limited.

To address the above concerns, the objectives of this paper are twofold: to determine the *K*_0_ value of compacted loess by using a triaxial apparatus, and secondly, to evaluate the effects of initial shear stress on the undrained response of compacted loess.

## Experimentation

### Test material and sample preparation

**Figure 2:**
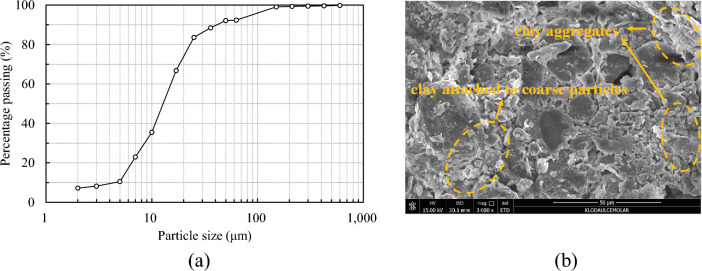
The particle size distribution curve and microscopic image of the test material: (**a**) particle size distribution curves of test materials; (**b**) microscopic image of compacted loess.

The loess used in the test was sampled in Yan'an City, Shaanxi Province, China, with an in-suit natural void ratio of 0.77 and a natural moisture content of 12%. Figure 2 shows the particle size distribution curve of the loess measured by sieving and sedimentation tests along with an image at the microscale, and it has a clay content of 10.5% by mass. As seen in Fig. 2b, two main forms of clay particles are visible in the image, namely clay-particle agglomerates and clay. In the former one, clays tend to assemble around the big particle with face contacts and point contacts, forming the interlocking pores. To evaluate the effect of clay content on *K*_0_, an extra amount of clay mainly composed of illite and montmorillonite was mixed with the loess, and the maximum clay content increases to 20%. Other physical properties of the test materials are also summarized in Table 1.

**Table 1:** Basic physical properties of test materials.

Test materials	Plastic limit (%)	Plasticity index (%)	*G*_s_	*d*_50_ (mm)	*d*_10_ (mm)
Loess	16.1	11.7	2.65	12.8	4.4
Clay	59	68	2.7	–	–

**Figure 3:**
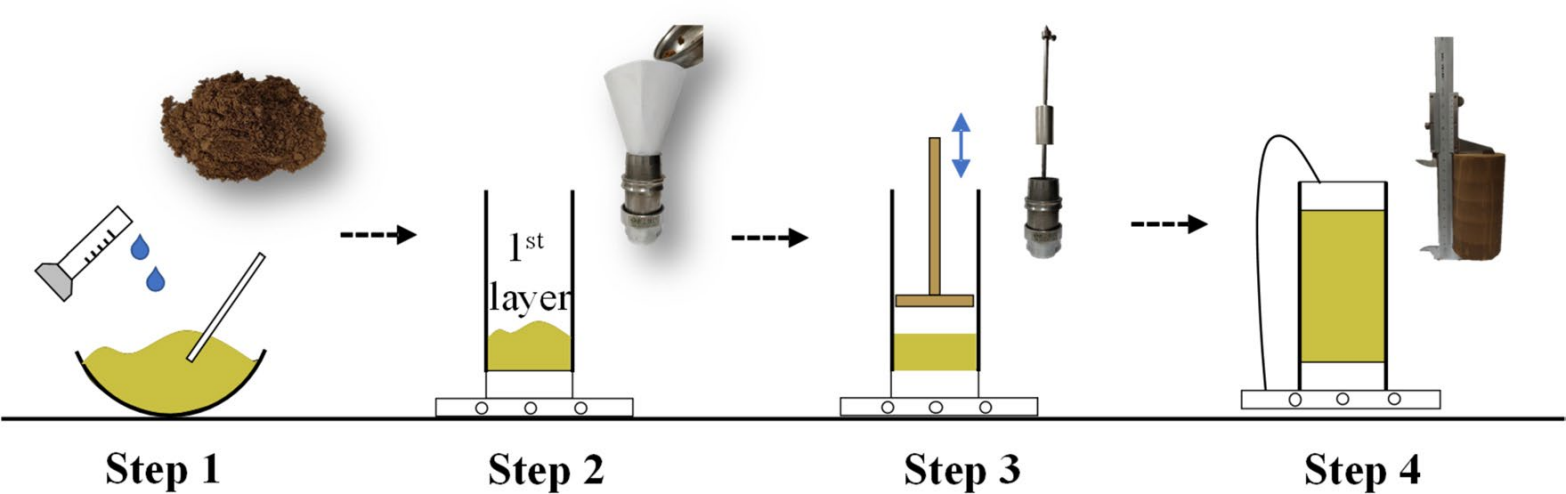
Schematic illustration of moist tamping method in sample preparation (Step 1: mixing soil at target water content; Step 2: transferring soil into a split mould by layers; Step 3: rough the surface of layers and compaction; Step 4: disassemble mould).

**Figure 4:**
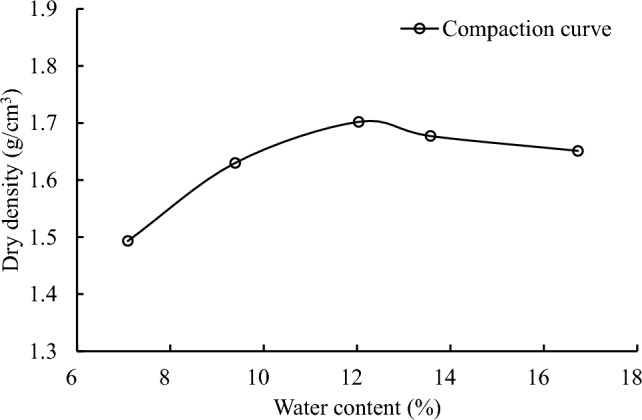
Loess compaction curve.

In this study, loess samples were prepared by the moist tamping (MT) method in conjunction with the under-compaction technique^20,21^. This method was selected because it is perhaps the best way to mimic the process of earth-fill project in the field, and it can produce high quality samples without segregation of particles^22^. As shown in Fig. 3, to obtain a target void ratio using the MT method, a pre-determined mass of wet soil was deposited into a split mold and then it was subjected to continuous tamping until the target height of the layer was achieved, and the degree of under-compaction was varied linearly from the bottom to the top layer, with an under-compaction ratio of 1%. In Fig. 4, the compaction curve of the tested loess is presented. Given that the optimal water content of the loess is around 12%, hence, an initial water content of 11.6% is at the dry side within 5% of the optimal water content, which is often required for the soil compaction in engineering practices.

### Test procedures

After the sample preparation, each loess sample was placed into a triaxial chamber and subjected to a saturation process. In this study, cylindrical samples of 50 mm in diameter and 100 mm in height were used in a triaxial apparatus. All samples were saturated in two stages: initially by flushing the sample with carbon dioxide^21,23,25^ and de-aired water, and then by applying a stepwise back pressure. To achieve the full saturation state, a Skempton *B* value (the ratio between an increment of confining pressure and the corresponding change in pore pressure) greater than 0.95 is required^24^.

**Figure 5:**
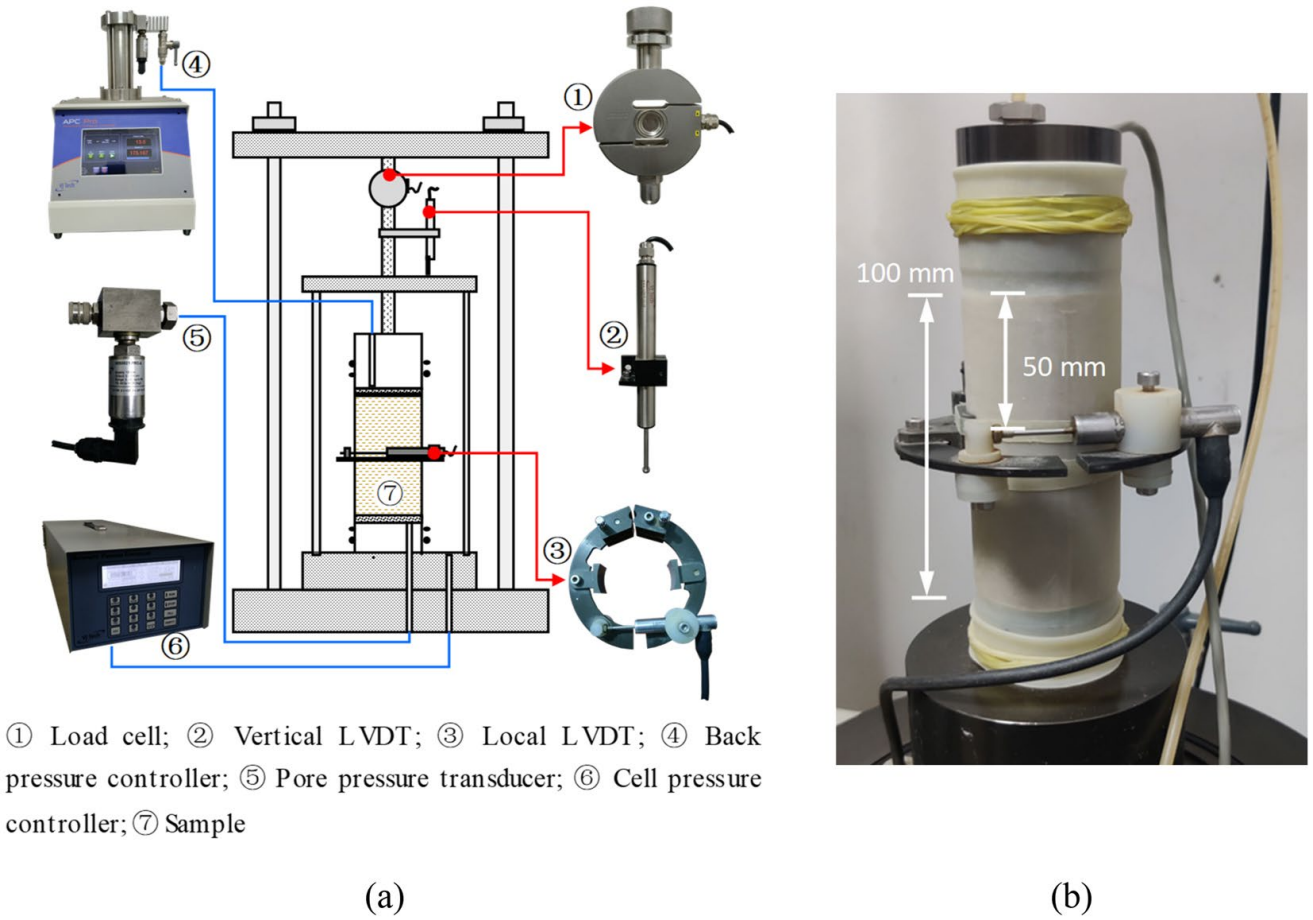
Detailed setup: (**a**) triaxial apparatus; (**b**) set up of local LVDT.

In this study, tests were conducted with an automatic triaxial testing system (Fig. 5). By adopting a *K*_0_ consolidation module in the triaxial system through a feedback control, the increment of deviatoric stress (*q*) was controlled with the premise that the volumetric strain (*ε*_v_) of the sample equals to the axial strain (*ε*_a_). Therefore, the samples can reach the *K*_0_ stress state progressively. Here, *ε*_a_ and *ε*_v_ were determined from the readings of vertical linear variable differential transformer (LVDT) and change of back volume, respectively. To bring the specimens to a target effective horizontal stress (*σ’*_3_), a slow loading rate of 3 kPa/hr was employed, thus the excess pore water pressure, if any, was very small and could be neglected. A local LVDT of high-precision (linear range of ± 2.5 mm with an accuracy of 1.6 μm) was mounted at mid-height position of the samples to check the *K*_0_ stress state as to whether no lateral strain (*ε*_r_) was developed. After reaching *K*_0_ stress state, however, the increments of radial deformation remained zero. The *K*_0_ consolidation stage was then followed by an undrained shear test. In order to ensure a clear response of pore water pressure in the shear stage, the strain rate was chosen to be 0.167 mm/min. For comparison, a series of tests were also carried out on isotropically consolidated loess samples (Table 2).

**Table 2:** Summary of *K*_0_ and isotropic consolidation on loess.

Test	Initial void ratio, *e*_0_	Consolidation conditions	Clay content (%)	*σ’*_3_ (kPa)	*p*’ (kPa)	After consolidation void ratio, *e*_c_	*K*_0_
K-1	0.775	*K*_0_ consolidation	10.5	100	132	0.747	0.51
K-2	0.596			100	186	0.584	0.28
K-3	0.768			150	195	0.712	0.53
K-4	0.772			250	352	0.707	0.45
K-5	0.772			350	499	0.695	0.44
K-6	0.594		15	100	157	0.587	0.36
K-7	0.597		20	100	133	0.591	0.49
R-1	0.732	Isotropic consolidation	10.5	195	195	0.721	–
R-3	0.745			132	132	0.737	

## Test results and discussions

### Validation of *K*_0_ stress state

It is noted that at the *K*_0_ stress state for soils, no lateral strain was developed^26^. In this study, to reach the *K*_0_ stress state, using membranes as the lateral confinement in the triaxial test is more challenging than using the conventional oedometer test with the rigid wall. Therefore, prior to address the shear behaviour of loess, a pressing concern is to examine whether the premise of no lateral strain is fulfilled in the *K*_0_ consolidation stage.

**Figure 6:**
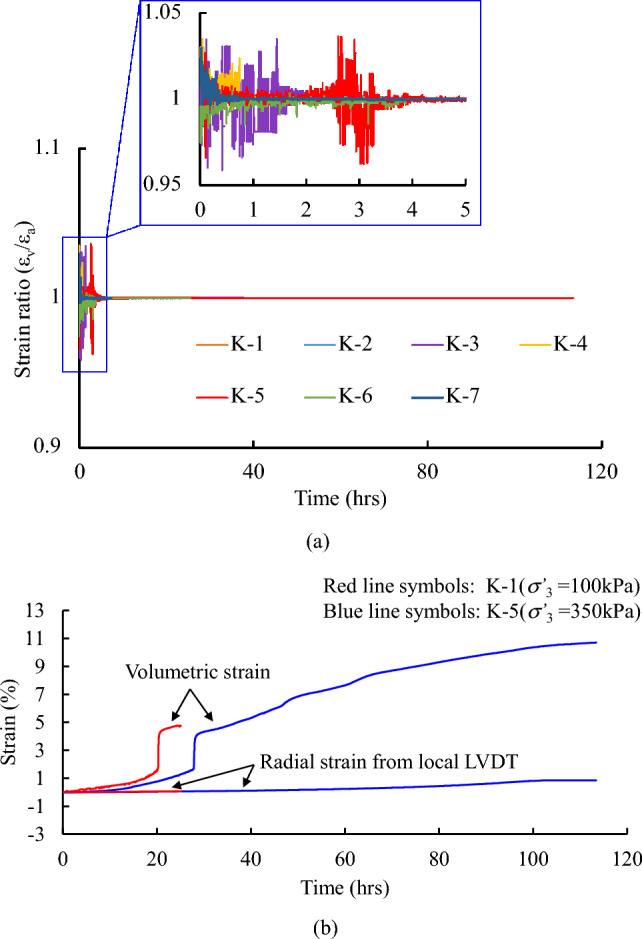
Measurements of strain in loess samples during *K*_0_ consolidation: (**a**) comparison of volumetric strain and axial strain; (**b**) comparison of radial strain and volumetric strain.

Figure 6a presents development of the ratio between the volumetric and axial strain with time. It is clear to see that a little fluctuation within a range of 0.95 and 1.05 occurs at the initial consolidation stage (4 h), and it is then followed by a steady trend with the ratio approaching to one. This finding implies that the lateral strain of the samples is not developed at the most of time in consolidation. Moreover, Fig. 6b plots the readings of the radial and volumetric strains with time for the specimens subjected to a target effective horizontal stress (*σ’*_3_ = 100 kPa and 350 kPa) in the consolidation stage. It is seen that the tendency in the development of the radial strain has flattened out with consolidation time. Compared with the maximum volumetric strain of approximately 8%, the development of maximum radial strain is relatively small (less than 0.8%). The above observations strongly indicate a satisfactory performance of the feedback control using the *K*_0_ module in the triaxial test system, such that a general validity to the *K*_0_ consolidation state for loess has been achieved.

### *K*_0_ consolidation path and *K*_0_ values

**Figure 7:**
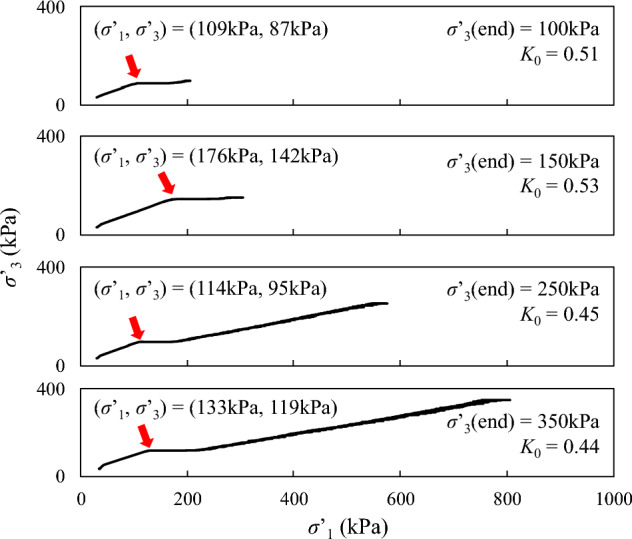
*K*_0_ consolidation path for loess samples at similar initial void ratios, *e*_0_ ≈ 0.775.

Figure 7 presents a set of test results in *K*_0_ consolidation for loosely compacted loess samples (*e*_*0*_ ≈ 0.775). It is seen that the change of effective horizontal stress (*σ’*_3_) is smaller than effective vertical stress (*σ’*_1_, *σ’*_1_ = *q* + *σ’*_3_) and the slope in the diagrams indicates the change of *K*_0_. Interestingly, a temporary plateau with a stepwise increment of* σ’*_3_ is consistently observed. An inflection point is marked in the diagram using a downward arrow. It appears that the inflection point is not dependent on the target effective horizontal stress. At a higher effective stress level after the plateau, if any, σ*’*_3_ continues to increase with *σ’*_1_. It should be noted that the *K*_0_ value at the end of consolidation is usually taken as the representative value, while others are nominal *K*_0_ values due to the effect of initial isotropic stress state^27^.

**Figure 8:**
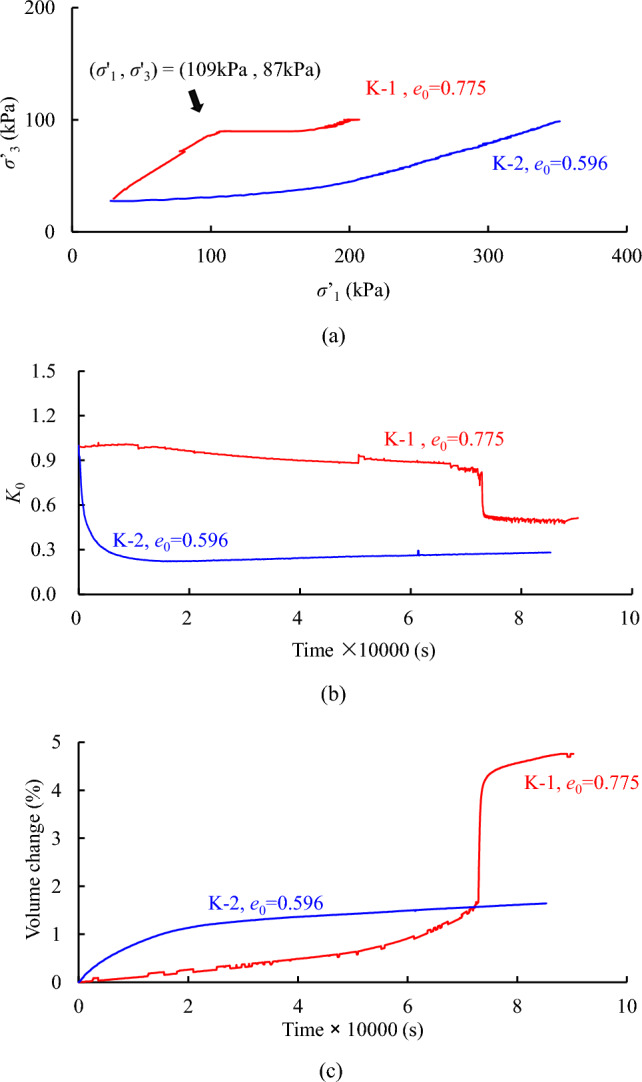
Comparison of *K*_0_ consolidation for loess samples with different initial void ratios.

*K*_0_ consolidation of loess samples prepared at two different packing densities are compared in Fig. 8. A marked difference in Fig. 8a is that the dense sample does not exhibit a stepwise increment of *σ’*_3_. Accordingly, the changes of *K*_0_ are revealed in Fig. 8b, showing that the *K*_0_ value starts to decrease from a reference isotropic stress state with the elapsed time. The *K*_0_ of the dense sample reduces as long as the load is applied and reaches a more or less constant value around 0.28, whereas the *K*_0_ of the loose sample mildly reduces until an abrupt reduction taking place around 20 h after applying the loads. Evidently, the *K*_0_ value is greater for the loose samples. Figure 8c shows the change of sample volume during the *K*_0_ consolidation. An unexpected volume change in sample K-1 is observed, that is also accompanied with the abrupt change in *K*_0_ value (Fig. 8b).

When we recalled from the fundamentals of soil mechanics, the observations in Fig. 8c are similar with the path for normal and over-consolidated soils. In brief, the initial path in K-1 is elastic and its slope is dictated by Poisson's ratio, while the latter part follows the plastic flow^28^. For a denser sample (K-2), the yield stress is greater. Hence, it is postulated that an increase of volume would occur, if a higher *σ’*_3_ is applied to the dense sample. In addition, an alternative explanation at the micro scale is perhaps due to an internal collapse of pores in the loose samples, and it contributes to the marked volume change^29^.

**Figure 9:**
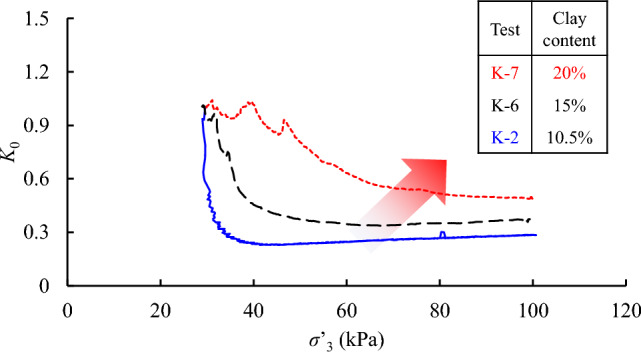
Comparison of *K*_0_ for loess samples with different clay contents.

Figure 9 presents the experimental results from the original loess and the loess-clay mixtures. Noting that the test materials in this diagram were prepared at the same initial void ratio (*e*_0_ ≈ 0.60) and the target effective horizontal stress (100 kPa), so that the differences in *K*_0_ value were solely attributed to the presence of additive clay. In general, the *K*_0_ value in this diagram decreases with an increment of *σ’*_3_. The reduction of *K*_0_ value is more severe in the original loess sample with lower clay content (K-2). In Table 2, the *K*_0_ values of test materials are summarized and it varies between 0.28 to 0.49 with different clay contents. Given a general trend of clay content that increases with loess distribution from north to south in the Chinese Loess Plateau^30^, caution should be exercised in engineering designs at different locations using loess.

### Undrained response analysis

**Figure 10:**
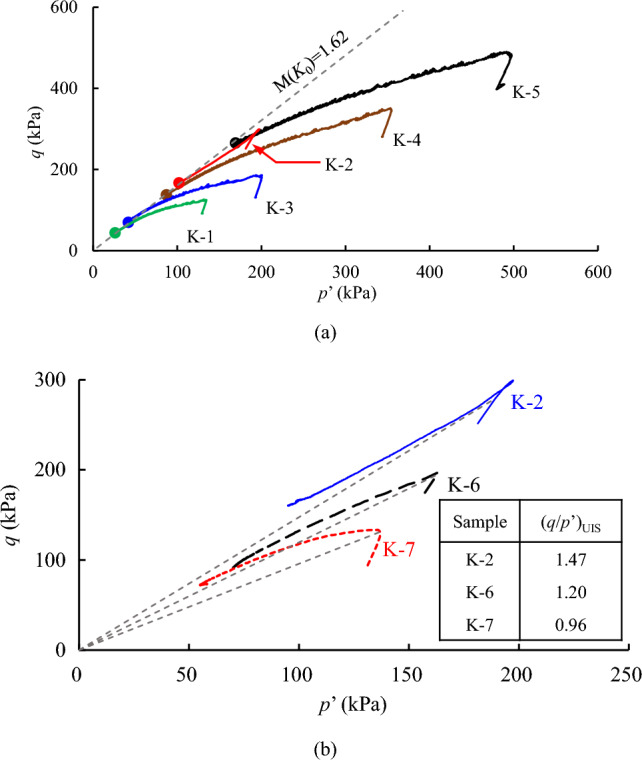
Undrained shear behaviour of loess subjected to *K*_0_ consolidation: (**a**) original loess; (**b**) loess-clay mixtures.

In Fig. 10a, results of stress path are presented in *q*-*p*’ diagram, and it demonstrates a contractive behaviour. Here, *p*’ is the mean effective stress (*p*’ = (*σ’*_1_ + 2*σ’*_3_)/3). Noteworthy is that “K-1” and “K-2” are at the same effective horizontal stress level (*σ’*_3_), yet the magnitudes of deviatoric stress at the start of shear stage are different. Known from the preceding analysis, it is attributed to the discrepancy of *K*_0_ (Table 2). The dense sample (K-2) yields a greater *q*. A straight line is used to connect the stress origin and the final stress state (ultimate axial strain is greater than 25%) of original loess sample, and it is also known as the critical state line (CSL) in *q*-*p*’ diagram^31,32^. It is seen that original loess specimens subjected to the *K*_0_ consolidation have a unique CSL and the slope of the line is 1.62. Besides, the undrained responses of loess-clay mixtures are compared in Fig. 10b. Evidently, the sample with higher clay content exhibits lower deviatoric stress at the start of shear stage, and the strength at the critical state is also smaller. Besides, the undrained unstable state (UIS) line that characterizes the onset of flow deformation is given in Fig. 10b. Here, the UIS line in the stress space (*q*-*p*’) is referred as the linear line passing through the peak point and the origin in each triaxial undrained shear test. In this plot, it can be seen that the slope angle of the UIS line, denoted as a stress ratio *q*/*p*’, increases with a reduction in the clay content.

**Figure 11:**
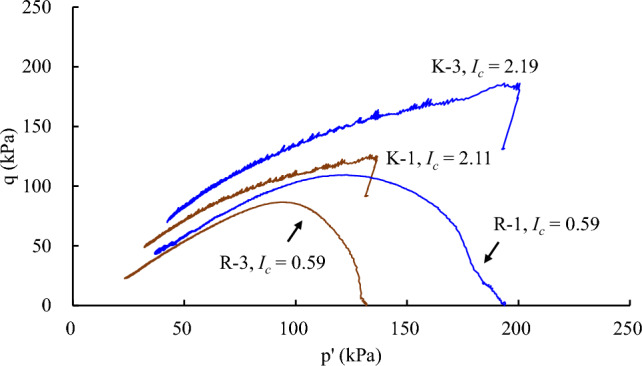
Undrained shear behavior of loess subjected to isotropic and *K*_0_ consolidation.

To examine the effect of *K*_0_ stress state on the undrained behaviour while isolating other possible influencing factors, in Fig. 11, test results are compared at similar post-consolidation void ratios (*e*_c_) and mean effective stress levels (*p*^’^) (R-1 vs. K-3; R-3 vs. K-1). At a given *p*^’^, the peak *q* is higher for the loess sample subjected to the *K*_0_ consolidation than that in isotropic consolidation. Besides, compared with the isotropic consolidated sample in Fig. 11, the *K*_0_ consolidated sample yields a slightly dilative behaviour (d*p*’ > 0) in the undrained condition at the beginning of the shear stage.

Here, the mean effective stress *p*’ is determined as follow,

1 $$p{\prime} = \frac{{2\sigma_{3}{\prime} + \sigma_{1}{\prime} }}{3}$$

where *σ*_1_’ is the major principal stress,* σ*_3_’ is the minor principal stress.

In the triaxial test, the following relationship can be obtained,

2 $$\sigma_{1}{\prime} = \sigma_{c} + q - u$$

3 $$\sigma_{3}{\prime} = \sigma_{c} - u$$

where *σ*_c_ the confining stress, *q* is the deviatoric stress, *u* is the pore pressure.

By combing the Eq. (1), (2) and (3),

4 $$p{\prime} = \sigma_{c} + \frac{q}{3} - u$$

**Figure 12:**
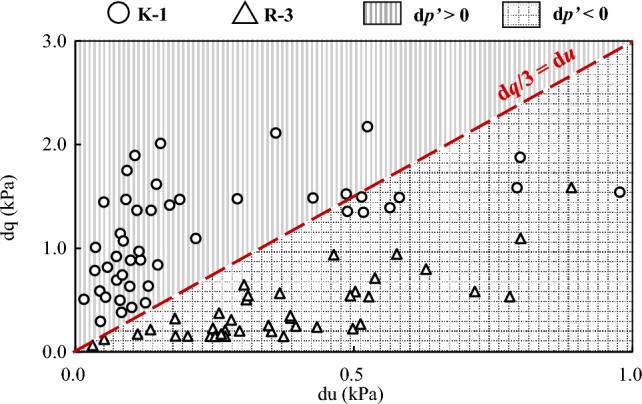
The relationship between pore pressure increment and deviatoric stress increment of loess.

When d*q* > 3d*u*, it can be derived that d*p*’ > 0. In Fig. 12, test results of d*q* and d*u* from the sample K-1 and R-3 are compared. It is noted that the slope of the diagonal line represents the ratio of d*q*/d*u*, and it equals to 3. Specifically, the diagonal line sets a benchmark. At the beginning of the shear test, the data from the *K*_0_ consolidated sample are located above the line, indicating a slightly dilative behaviour with d*p*’ > 0. Under otherwise similar conditions, the data for the isotropic-consolidated sample are located below this line, indicating a contractive behaviour. The findings in Fig. 12 provide a rational explanation on discrepancies of the effective stress path between the isotropic consolidated sample and the *K*_0_ consolidated sample in Fig. 11.

To quantify the degree of strain softening with reference to the initial static shear stress, a parameter *I*_c_, termed the modified collapsibility index, is introduced, following the concept of Sivathayalan and Vaid^33^as follows.

5 $$I_{C} = \frac{{q_{peak} - q_{{{\text{cs}}}} }}{{q_{peak} - q_{0} }}$$

where *q*_cs_ and *q*_0_ are the deviator stress at the critical state and prior to the undrained shearing, respectively. When *I*_c_ = 0, it corresponds to no strain softening and the soils would exhibit completely dilative behaviour. When *I*_c_ is greater than one, it implies the critical state strength is lower than the initial static shear stress, and thus the triggering of a flow slide if equilibrium is disturbed by a small undrained perturbation. As indicated in Fig. 11, at a similar state in terms of the void ratio and the mean effective stress level, *I*_c_ is consistently higher in the *K*_0_ consolidated samples than in the isotropic consolidated ones. This observation implies that the *K*_0_ consolidated loess samples are more susceptible to severe failures.

## Conclusions

This paper aims to not only characterize the *K*_0_ of compacted loess, but also to explore the undrained response with the presence of initial shear stress by using the triaxial test. Several major findings from the experimental results are summarized as follows: (1) the *K*_0_ of loess samples is density-dependent. A distinguishable decrease of *K*_0_ value is revealed in the course of *K*_0_ consolidation for the relatively loose sample; (2) the increase of clay content in loess induces higher *K*_0_ value; (3) *I*_c_ is consistently higher in the *K*_0_ consolidated samples, which implies that the *K*_0_ consolidated samples are more susceptible to severe failures. This study suggests a pressing need to consider the *K*_0_ value of compacted loess in a rigorous manner in geotechnical designs, and the associated impacts on the undrained response of loess should be taken into accounted properly.

## Data Availability

The datasets used and/or analysed during the current study available from the corresponding author on reasonable request.
